# How to “inoculate” against multimodal misinformation: A conceptual replication of Roozenbeek and van der Linden (2020)

**DOI:** 10.1038/s41598-023-43885-2

**Published:** 2023-10-25

**Authors:** Julian Neylan, Mikey Biddlestone, Jon Roozenbeek, Sander van der Linden

**Affiliations:** 1 TILT, The Hague, The Netherlands; 2 Department of Psychology, University of Cambridge, Cambridge, UK

**Keywords:** Human behaviour, Health policy

## Abstract

Building misinformation resilience at scale continues to pose a challenge. Gamified “inoculation” interventions have shown promise in improving people’s ability to recognize manipulation techniques commonly used in misinformation, but so far few interventions exist that tackle multimodal misinformation (e.g., videos, images). We developed a game called *Cat Park*, in which players learn about five manipulation techniques (trolling, emotional manipulation, amplification, polarization, and conspiracism), and how misinformation can spread through images. To test the game’s efficacy, we conducted a conceptual replication (*N* = 380) of Roozenbeek and van der Linden’s 2020 study about *Harmony Square*, with the same study design, item set, and hypotheses. Like the original study, we find that people who play *Cat Park* find misinformation significantly less reliable post-gameplay (*d* = 0.95, *p* < 0.001) compared to a control group, and are significantly less willing to share misinformation with people in their network (*d* = 0.54, *p* < 0.001). These effects are robust across different covariates. However, unlike the original study, *Cat Park* players do not become significantly more confident in their ability to identify misinformation (*p* = 0.204, *d* = − 0.13). We did not find that the game increases people’s self-reported motivation and confidence to counter misinformation online.

## Introduction

Misinformation continues to pose a problem and has been implicated in impacting democratic processes^1–3^, climate change perceptions^4–6^, and public health outcomes^6–8^. Although we recognize scholarly debate on how to best conceptualize the concept of misinformation^3,9^, we here define misinformation here as any kind of false or misleading information (the latter may also include information that distorts facts, is logically fallacious or stripped of relevant context)^10^. We define political misinformation as misinformation intended to achieve a political aim.

Recent reviews of how to effectively counter the spread of misinformation^3,10,11^ have identified prebunking, or preemptive debunking, as a promising method to confer resilience against misinformation^12^. Prebunking is commonly grounded in psychological inoculation theory. Inoculation theory was developed by William McGuire in the 1960s^13–16^, and posits that by exposing people to psychological persuasion techniques that may be used against them, you can preemptively “inoculate” people against further attempts to use those same persuasive techniques, much like how a vaccine exposes patients to a weakened version of a virus in order to train the body’s immune system to respond to an infection. So-called “technique-based” (also called “logic-based”) inoculation interventions focus on the manipulation techniques and tactics that commonly underlie misinformation, rather than individual examples only specific to certain contexts^17,18^. Several reviews and meta-analyses of inoculation theory have supported its potential efficacy in building public resilience against misinformation^5,10,12,19–21^.

In collaboration with the Dutch organization Tilt, design agency Gusmanson, the U.S. Department of State’s Global Engagement Center and the US Embassy in the Hague, we created a 15-min, free online browser-based game called *Cat Park*. The game builds on other gamified misinformation interventions such as *Bad News*^22^, *Harmony Square*^23^, *Go Viral!*^24^, and *Cranky Uncle*^25^*.* In each of these games, the player develops psychological resistance against common forms of online manipulation by being exposed to less potent doses of these techniques in a controlled environment.

*Cat Park* differs from these games in that it focuses on the multimodal elements of online manipulation. Most research on misinformation has focused on textual content, whereas multimodal misinformation (e.g., videos, images) has remained underexplored^26^. In particular, interventions against visual misinformation are lacking as social media platforms have invested predominantly in text-based AI moderation, which does not address multimodal threats^27^.This is a major gap in the literature because videos and images often spread further than text alone^28,29^. Access to media editing tools has been steadily expanding, allowing anyone with internet access to alter images with increasingly sophisticated tools such as image-to-image translation using machine learning^30^. Visual misinformation can also be more difficult to address because it contains “an implicit guarantee of being closer to the truth than other forms of communication”^31^ in other words, people perceive visual misinformation to be more credible than textual misinformation^28^.

## What is Cat Park?

*Cat Park* is an interactive game about controlling the narrative about a city government project (the building of a cat park). In the game, the player takes on the role of someone new in town. They are shown around the city by four different characters, and complete tasks to foment discontent over the building of a cat park. A central theme of the game is that misinformation, even when used for a morally correct cause (i.e., reducing potentially wasteful municipal funding on a cat park), can have destructive consequences. Over the course of the game, the player develops a following, creates conspiracies, and exacerbates tensions between groups (people in favor of the cat park and those against it). In doing so, players are exposed to 5 common manipulation techniques (the same as those from the *Harmony Square* game^23^:

1. Trolling, i.e., provoking people to react emotionally, which evokes anger^22,32^.
2. Exploiting emotional language, i.e., using emotional appeals to make people angry or afraid about a topic or issue^31,33^.
3. Amplifying the reach and popularity of certain messages through maximizing the shareability of messages^34,35^.
4. Developing conspiracy theories, i.e., subjectively attributing power to supposedly clandestine and nefarious groups to blame them for world events^35–38^.
5. Polarizing audiences by deliberately emphasizing and magnifying inter-group differences^2,39^.

In addition to these five techniques, *Cat Park* players also learn about how multimodal misinformation in the form of modular memes and altered images is created^26^. There are more disinformation techniques than the ones included in the game, however we opted to select 5 techniques that are prevalent in the literature^12,18,22,23^. The game features 3 interactive mini-games that serve to expose players to techniques used to spread multimodal misinformation. In the first mini-game, the player alters existing images to create evidence for their conspiracy theories. In the second, the player is given text prompts and images and tasked with creating memes to amplify their narrative. In the third, the player must identify components of an image that have been altered such as duplicated elements and the incomplete erasure of portions of the original image. By exposing people to these manipulation techniques in a safe environment and prompting them to refute these techniques, the game presents people with a “weakened dose” of misinformation, in line with inoculation theory^16^.

**Figure 1:**
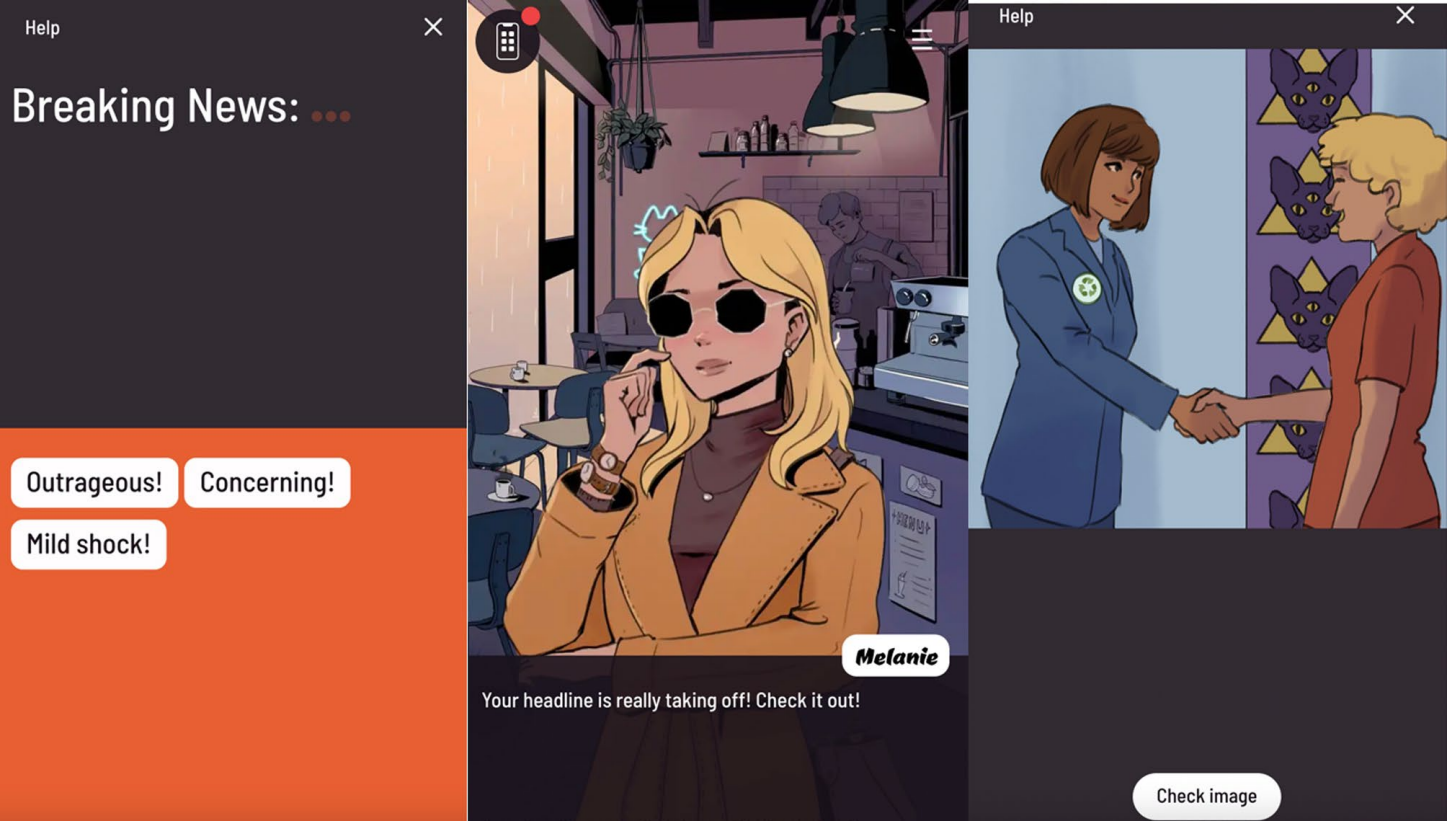
Screenshots from the Cat Park game.

The final outcome of the game is the violent destruction of the cat park. Unlike *Harmony Square*, where players are left to ponder the consequences of their actions, *Cat Park* encourages players to make amends and try to mitigate the consequences of their actions. They are prompted to try to counter their own online manipulation campaign by taking countermeasures such as fact checking. However, they are also warned that these countermeasures may not always be effective for all internet users^32^, for example, because people frequently continue to believe falsehoods even after they have been debunked^36,40^, a phenomenon known as the “continued influence effect” (Fig. 1).

## The present study

To test the efficacy of *Cat Park* as a way to “inoculate” people against various forms of misinformation, we conducted a conceptual replication of the *Harmony Square* study by Roozenbeek and van der Linden^23^. We utilized the same experimental design and methodology as Roozenbeek and van der Linden in their study on *Harmony Square*^23^*.* We showed the participants in our study 16 social media posts. These social media posts are the same as those we used to evaluate the efficacy of *Harmony Square* and each of which made use of one of 5 manipulation techniques we exposed players to while playing *Cat Park*: trolling, using emotional language, amplification of messages, conspiratorial reasoning, and group polarization. We conducted a 2 (treatment vs control) by 2 (pre vs post) mixed design randomized controlled trial to test if *Cat Park* improved people’s ability to identify manipulative online content. We posited the same hypotheses as Roozenbeek and van der Linden^23^:

1. Participants in the experimental condition will find manipulative social media content significantly less reliable than the control group after playing.
2. Participants in the experimental condition will be more confident in their ability to identify manipulative content than the control group after playing.
3. Participants in the experimental condition will be less likely to indicate that they would be willing to share manipulative social media content within their network than those in the control group after playing.

To answer each of these questions, we replicated the methods of the *Harmony Square* study by first calculating the difference between the average score on all 3 of the above questions for all 8 “real misinformation” and all 8 “fictional misinformation” social media posts that we used as measures before and after the intervention, for each respondent. *Cat Park* has significant overlapping inoculation aims with *Harmony Square,* but includes a critical difference in its emphasis on multimodal misinformation.

**Figure 2:**
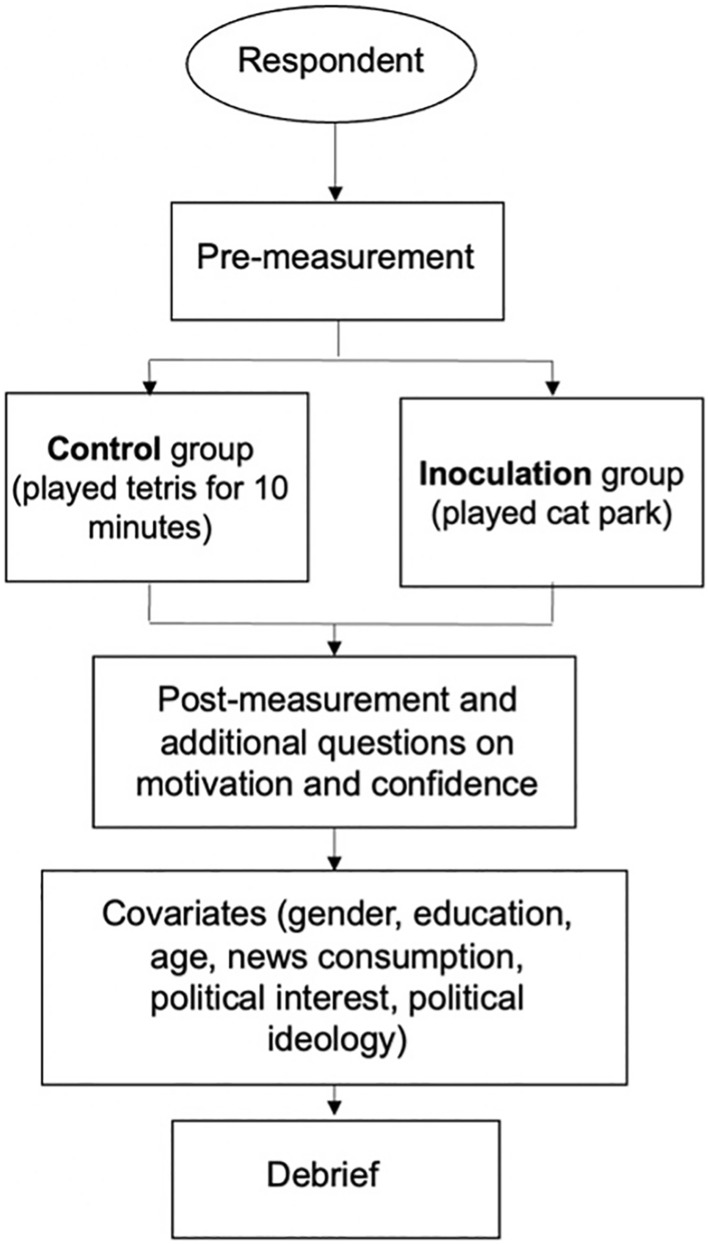
Experimental design flowchart.

We included three additional questions separate from the original study that were designed to primarily assess respondents' (1) general confidence in their ability to identify manipulated images, and secondarily confidence in their ability to identify both (2) emotionally manipulative online content, and (3) instigators of arguments online. Additionally, as there was an additional section on countering misinformation that was not featured in Harmony Square we asked how motivated participants felt to counter misinformation when they saw it, to determine whether the inoculation successfully motivated psychological resistance against misinformation. See Fig. 2 for an overview of the study design.

## Results

First, we conducted independent samples *t*-tests to determine whether the experimental manipulation (i.e., condition: inoculation vs. control) significantly increased motivation to counter misinformation online. The experimental manipulation did not confer significantly stronger psychological motivation in the treatment group compared to the control group (*p* = 0.451). Similar non-significant differences were also found for confidence in the ability to identify both emotionally manipulative online content and instigators of arguments online (both *p*s > 0.469).

**Figure 3:**
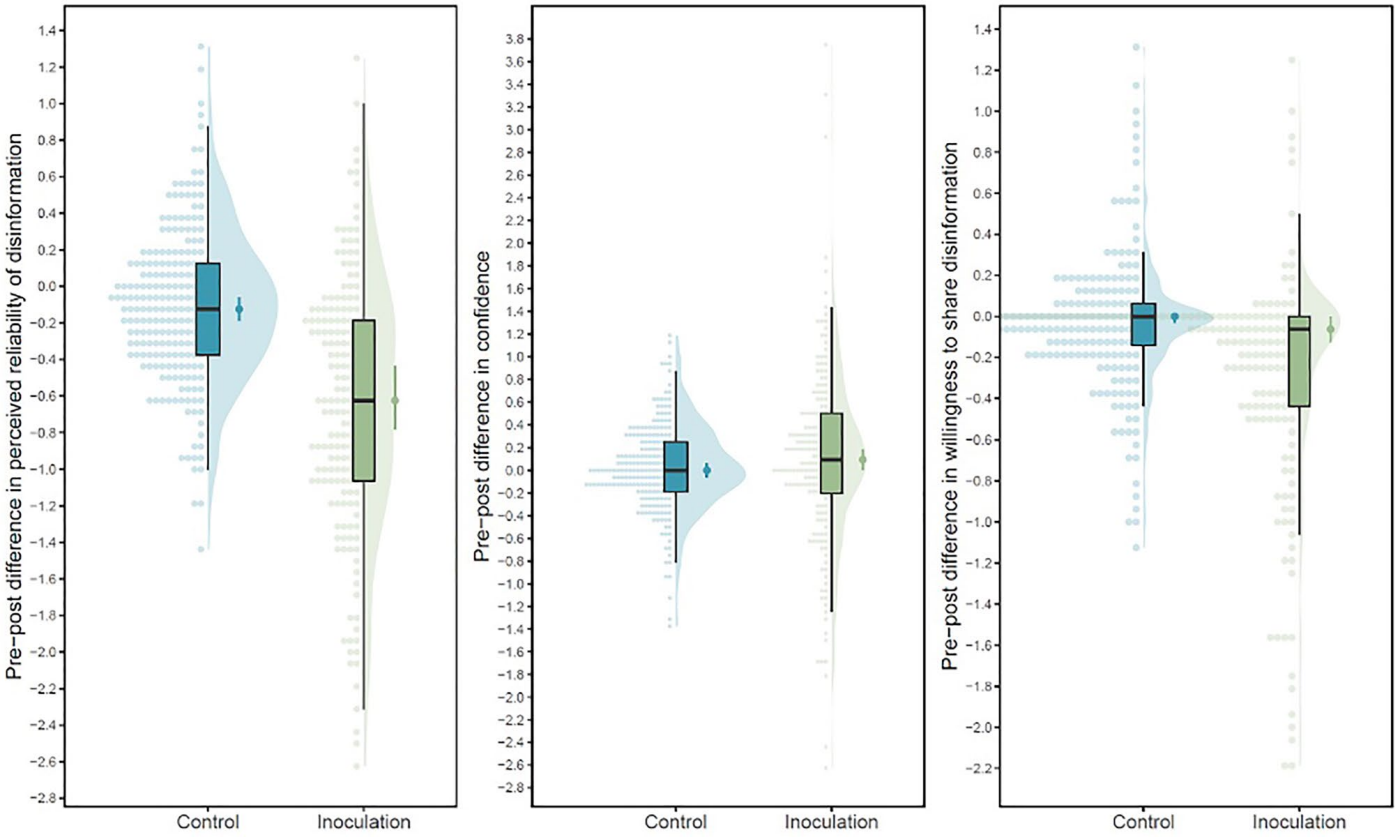
Box plots and point range plots with violin plots and data jitter for the pre-post difference scores for the reliability (top left), confidence (top middle), and sharing (top right) measures, for both the inoculation (Cat Park) and control (Tetris) groups.

**Figure 4:**
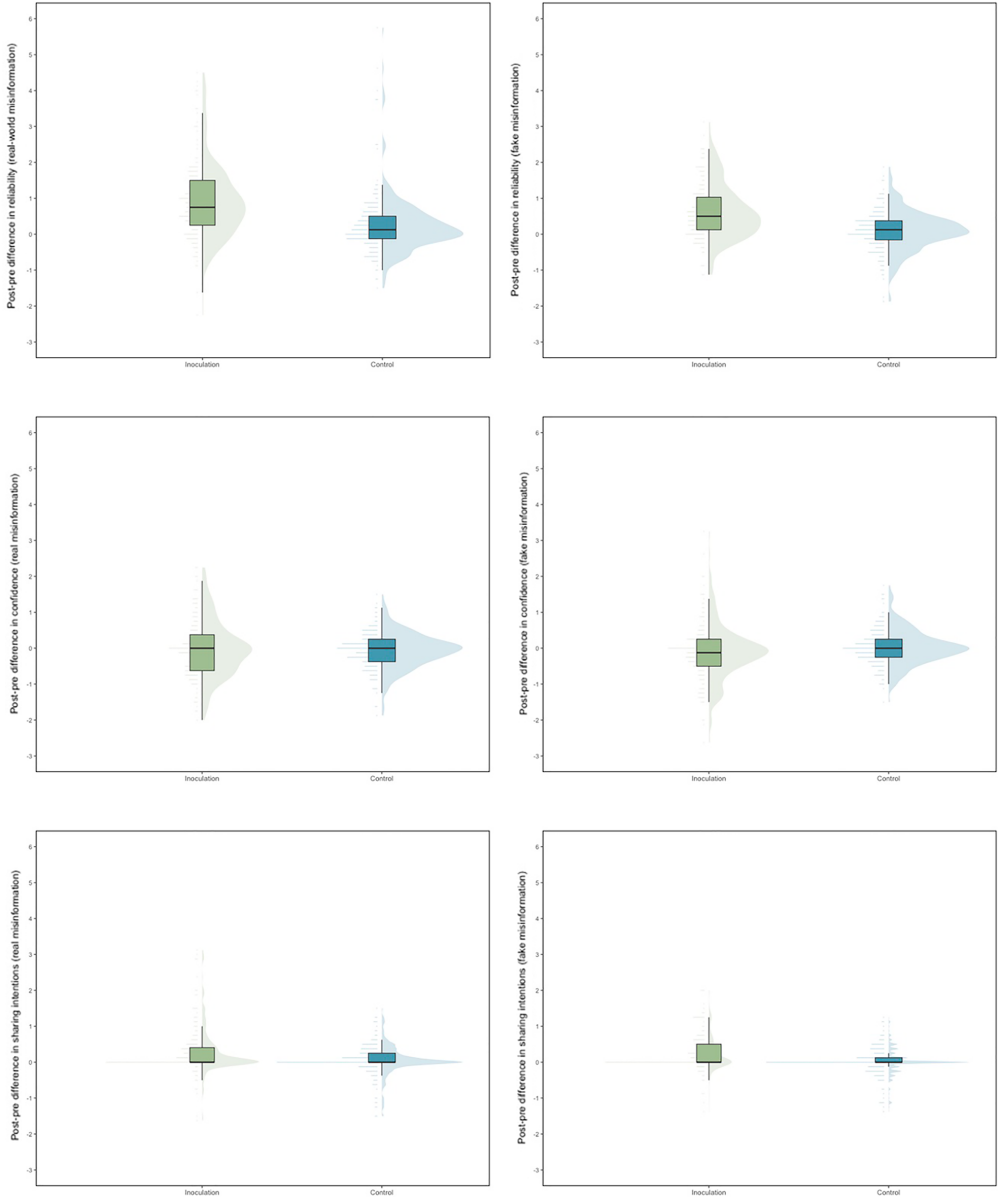
Box plots and point range plots with violin plots and data jitter for the pre-post difference scores for the perceived reliability of real misinformation (top left), perceived reliability of fake misinformation (top right), confidence in detecting real misinformation (middle left), confidence in detecting fake misinformation (middle right), sharing intentions of real misinformation (bottom left), and sharing intentions of fake misinformation (bottom right) measures, for both the inoculation (Cat Park) and control (Tetris) groups.

Next, we conducted independent samples *t*-tests to check if improvement in the other outcome variables (i.e., perceived reliability of misinformation, confidence, and sharing intentions) was significantly greater in the treatment group than in the control group. Exploratory confirmatory factor analyses revealed that model fit was acceptable when treating these outcome variables as single factors or when distinguishing between post type (real vs. fake) or manipulation technique (see Supplement). These analyses indicated that the reduction in perceived reliability of misinformation was significantly stronger in the treatment group, *M*_*Treatment*_ = − 0.65, *SD*_*Treatment*_ = 0.71, compared to the control group, *t*(380) = 9.20, *p* < 0.001, *M*_*Control*_ = − 0.11, *SD*_*Control*_ = 0.43, and this difference was large, *d* = 0.95 (see Fig. 3). An exploratory mixed ANOVA confirmed no interaction of this effect by post type (i.e., real vs. fake; see Fig. 4). Similarly, the reduction in willingness to share misinformation was significantly stronger in the treatment group, *M*_*Treatment*_ = − 0.27, *SD*_*Treatment*_ = 0.55, compared to the control group, *t*(380) = 5.21, *p* < 0.001, *M*_*Control*_ = − 0.04, *SD*_*Control*_ = 0.33, and this difference was medium in magnitude, *d* = 0.54 (see Fig. 3). An exploratory mixed ANOVA indicated that while this effect was significant regardless of post type (i.e., real vs. fake), the reduction in sharing intentions was significantly stronger overall for real posts, M_real_ = 0.17, SD_real_ = 0.57, than fake posts, M_fake_ = 0.12, SD_fake_ = 0.46, t(379) = 2.31, p = 0.022, but this difference was very small in magnitude, *d* = 0.12 (see Fig. 4). Finally, the change in confidence to detect misinformation was not significantly different between the treatment, *M*_*Treatment*_ = 0.11, *SD*_*Treatment*_ = 0.86, and control groups, *M*_*Control*_ = 0.02, *SD*_*Control*_ = 0.40, *t*(380) = 1.27, *p* = 0.204, *d* = − 0.13 (see Figs. 3, 4).

To check the robustness of our findings, we created an exploratory linear regression model with perceived reliability of misinformation, confidence in detecting misinformation, and willingness to share misinformation as simultaneous dependent variables, and experimental condition as the independent variable. Furthermore, we controlled for age, gender, education, political ideology, political interest, and both general and social media news consumption on all paths as covariates. In this model, the experimental manipulation remained a significant predictor of both perceived reliability of misinformation and willingness to share misinformation (see Supplement, Figure S1). With regards to the covariates, willingness to share misinformation was marginally predicted by lower education (*p* = 0.052) and significantly predicted by being male (vs. female; *p* = 0.023; see Supplement, Table S1). Additionally, we performed three separate dominance analyses to determine whether the experimental manipulation accounted for the largest amount of variance in the respective outcome variables. The experimental manipulation was confirmed to explain the largest amount of variance when compared with the covariates for perceived reliability of misinformation, *R*^2^ = 0.84, willingness to share misinformation, *R*^2^ = 0.52, and confidence in countering misinformation online, *R*^2^ = 0.30.

## Discussion

In this conceptual replication of the *Harmony Square* study by Roozenbeek and van der Linden^23^, we found that *Cat Park* players showed stronger reductions in perceptions of misinformation as reliable, and were less willing to share misinformation with others, compared to the control group. Importantly, the effect sizes for these findings overall are medium-to-large, are robust when controlling for covariates, and the intervention effect for reliability judgements (*d* = 0.95) is similar to that of the study that we replicated (*d* =  ~ 0.50 for the *Harmony Square* game). This effect size is consistent with (and even descriptively larger than) previous gamified interventions^21–25^, demonstrating that active inoculation against misinformation is an efficacious strategy to mitigate the spread of misinformation online.

In Roozenbeek and van der Linden’s *Harmony Square* study, respondents showed increased confidence in their ability to identify misinformation compared to the control group, but this finding was not present in players of *Cat Park*. This result follows Harrop, Roozenbeek et al.^41^, who also did not find an effect of another gamified inoculation intervention on attitudinal certainty (confidence), although they did find an effect for perceived reliability. This finding may be explained in several ways. First, although both games cover similar topics, they have different storylines and graphical presentations. Furthermore, *Cat Park,* unlike *Harmony Square,* features photo manipulations that people may feel are difficult to discern in reality (therefore not increasing their confidence). Indeed, studies have found that exposure to manipulated visual content makes people uncertain^42^. From a psychological perspective, the improved ability to effectively appraise the reliability of misinformation without improved confidence shown here may reflect the detection of unconscious knowledge^43^. That is, while our findings appear to demonstrate improved ability to detect misinformation as a result of the intervention, participants did not appear to be more aware of this ability post-gameplay. Recent evidence suggests that susceptibility to misinformation may, in part, be related to overconfidence in one’s beliefs and an unwillingness to admit when you are wrong (see Roozenbeek et al.^44^. Therefore, Lees et al's.^45^ demonstration that improved detection of manipulation techniques (in their case, trolling) can occur alongside reduced self-efficacy in detecting reliable information in one’s environment may reflect improved intellectual humility. Future research may focus on unpacking this phenomenon further.

Unlike previous interventions, *Cat Park* featured a section where players had to counter misinformation. We asked participants if the game made them more motivated to counter misinformation online, but did not find that those in the treatment group reported a stronger motivation than those in the control group. We included a measure of motivation as we wanted to determine if respondents would be more likely to counter disinformation after playing the game and outside of the study. Despite the central tenet of inoculation theory to motivate psychological resistance to misinformation, serious games are not always conducive to instilling motivation within players^46^. Additionally, our sample was self-selected within the Prolific environment, meaning participants may have had higher prior baseline motivation surrounding the topic than the general population, potentially making it harder to detect improvements due to ceiling effects^47^.

While our findings demonstrate a generally promising conceptual replication of the use of gamified inoculation to confer psychological manipulation against multimodal misinformation, there are a number of limitations. First, without the measurement of real news detection for comparison, we cannot be certain that participants in the treatment group did not simply find all information unreliable or unworthy of sharing, regardless of its objective veracity. Importantly, a recent preprint^48^ detailed a re-analysis of the efficacy of the Bad News game to improve the detection of both reliable and unreliable content, suggesting that it could simply increase overall skepticism of content of ambiguous reliability rather than reducing misinformation susceptibility specifically—though the variation in results, the lack of balanced and validated real news items, the presence of some bias and manipulation in real news stories, and opposite findings from a recent meta-analysis makes any definitive conclusions about response bias challenging^21,48^. Thus, future research could include standardized measures such as the Misinformation Susceptibility Test^49^ to further investigate this notion in the context of Cat Park. However, we would stress that some additional skepticism to manipulative techniques whether in true or false news can be a positive outcome in reducing people's susceptibility to influence overall.

Second, warranted concerns could be raised about order effects with regards to the repetition of test items both before and after the intervention was administered. That is, participants could have potentially familiarized themselves with the content of each item, displaying demand characteristics based on their assumptions around what was being measured. However, work by Maertens et al.^50^ examined potential learning or demand effects of repeated items in the Bad News game. Importantly, they found no difference in responses when pre- vs. post-test items were repeated or not. Furthermore, Mummolo and Peterson^51^ carried out experiments demonstrating that demand effects were not overtly present in surveys, even when the intent of the researcher was made explicit. Therefore, while we welcome future work focusing on how the Cat Park game might influence reliability judgements of real information, we believe that concerns around demand characteristics in this context are likely to be minimal.

Third, we did not administer the video content as part of our multimodal media stimuli because at present cruder image manipulations are more common than convincing manipulated videos^27^. While this still allowed us to replicate inoculation effects in the novel domain of images, future research would benefit from uncovering the parameters to psychological resistance against misinformation in video format. Additionally, the game included inoculation against five manipulation techniques and we recognize that real disinformation can draw from multiple techniques sometimes used simultaneously. Future studies should examine the effects of additional disinformation techniques as well as how they influence each other when used simultaneously. Finally, only native English-speakers were included in the data collection. Issues surrounding the lack of data on misinformation susceptibility and conspiracy beliefs in less Western, educated, industrialized, rich, and democratic nations have been widely discussed^50,51^. To further demonstrate the efficacy of this intervention, scholars and institutions should seek to collect similar data from these populations.

In this study, we tested the efficacy of a novel serious game, *Cat Park,* as a means to inoculate people against multimodal misinformation*.* While our study participants did not show improved confidence in their abilities, their effective appraisal of the reliability of image-based misinformation was improved, and they were less likely to indicate wanting to share it online when compared with the control group. These findings serve as a mostly successful conceptual replication of a previous study testing the use of serious games to inoculate people against online misinformation, further showing that this approach can also be used to improve detection of image-based misinformation. These findings may inform future efforts to mitigate the spread of various kinds of misinformation online. Cat Park will be publicly available in English, French, Dutch and Russian.

## Methods

We conducted a 2 (treatment vs control) by 2 (pre vs post) mixed design randomized controlled trial to test if *Cat Park* improved people’s ability to identify manipulative online content. We recruited participants (*N* = 380) from the online platform Prolific. The sample was an international sample of English-speaking countries (excluding the US). Ethics approval for the original study that we replicated conceptually here Roozenbeek and van der Linden^23^ was obtained under PRE.2020.052 (Cambridge Psychology Research Ethics Committee). The experiment was conducted in accordance with the ethical considerations of the original study. Informed consent was obtained from all participants. See Table S2 for the sample composition.

The treatment condition played *Cat Park* from beginning to end. The control condition played *Tetris* for 10 min. We utilized *Tetris* because it is in the public domain, it does not require extensive explanation, and it involves about the same amount of cognitive effort as playing *Cat Park*. *Tetris* has been used as a control to validate other digital game-based learning projects^22,24,45^.

In total 199 respondents were in the treatment group. However, only 181 were included in the final sample because a number did not fill in any information for the survey. We assessed whether respondents had completed the game by asking them which location was the last location they visited within the game. 199 respondents were in the control group which played *Tetris*, for a total sample of *N* = 380. We sought to test the same three hypotheses tested by Roozenbeek and van der Linden^23^:

**H1:** Playing *Cat Park* reduces the perceived reliability of social media posts that make use of manipulation techniques.

**H2:** Playing the game increases people’s confidence in their ability to spot such manipulation techniques in social media content.

**H3:** Playing the game reduces people’s self-reported willingness to share manipulative social media content with people in their network.

**Figure 5:**
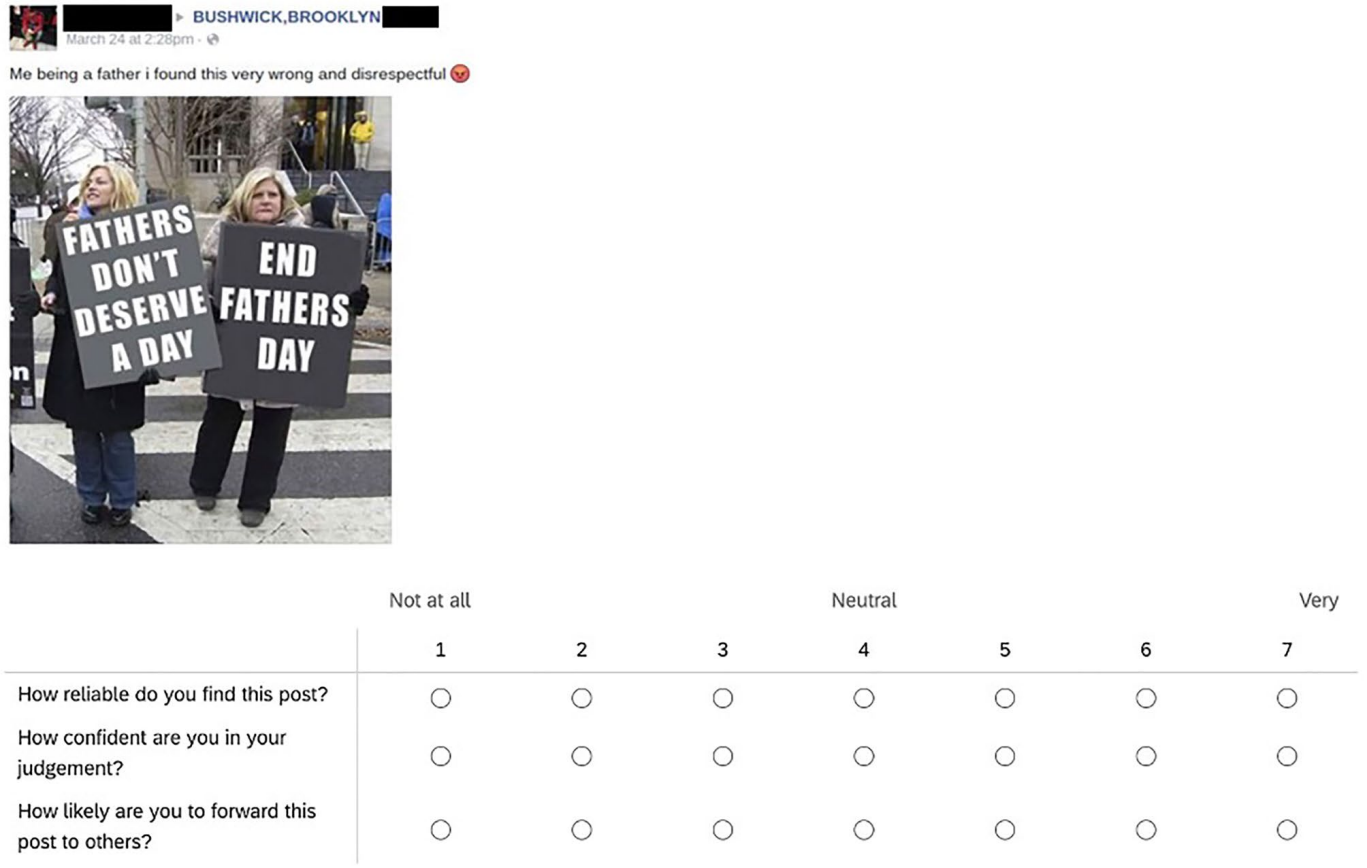
Example of a social media post used in the survey, along with the outcome measures (reliability, confidence, and sharing intentions).

To test these hypotheses, we administered the same item rating task before and after playing either *Cat Park* or *Tetris*, which consisted of rating 16 social media posts (i.e., the stimuli). These stimuli are the same as those used to evaluate the efficacy of *Harmony Square* and each of which made use of one of 5 manipulation techniques learned while playing *Cat Park*: trolling, using emotional language, amplifying the reach of messages, conspiratorial reasoning, and group polarization. Aside from the *Harmony Square* study^23^, some of these stimuli were used in a previous study by Traberg and van der Linden^52^. Underneath each post, participants were asked three questions (on a 1–7 scale ranging from “not at all” to “very”) which served as our primary outcome measures of interest: how reliable do you find this post? (reliability); how confident are you in your judgment? (confidence); and how likely are you to forward this post to others? (sharing). See Fig. 5 for an example of one of the stimuli plus each of the outcome measures.

The stimuli contained both politically partisan and politically neutral content. The political content had an equal number of right-leaning and left-leaning items. Our survey contained the same 8 posts of “real” manipulative content pulled from social media and 8 “fictional” social media posts that were used in Roozenbeek and van der Linden^23^. As in Roozenbeek and van der Linden^23^ we did not hypothesize any significant differences between participants’ assessments of “real” (i.e., examples of misinformation that went viral online) and “fictional” (i.e., stimuli that were invented by the researchers) misinformation, but chose to include both types because:

1. including “real” items increases the external validity of the study, as participants are tested on information that they could have encountered in their own social media feeds;
2. including “fictional” items maximizes experimental control and thus allows us to better isolate each manipulation technique and ensure political neutrality, and
3. By including “fictional” items, we account for the possibility that participants may have seen the “real” manipulative content before, a memory confound which could bias their assessment^22^.

Following Roozenbeek and van der Linden^23^, we deliberately chose to only include manipulative content, as opposed to having both manipulative and non-manipulative content. The purpose of both *Cat Park* and *Harmony Square* was not to have players learn how to distinguish between high-quality and low-quality content, but instead to teach people how to spot common types of misinformation techniques on social media. For both studies the aim is to assess whether the interventions were effective at reducing susceptibility to political misinformation, rather than truth discernment^53^. However, we acknowledge that media literacy interventions can affect the rating of both credible and non-credible items^54–56^.

Aside from this item rating task, we also administered a series of questions related to participants’ motivations to counter misinformation online. Specifically, we asked the following questions (on a 1–7 scale ranging from “not at all” to “very”): *“How motivated do you feel to counter misinformation when you see it?”; “How confident do you feel in your ability to identify manipulated images on social media?”; “How confident do you feel in your ability to identify emotionally manipulative online content?”;* and *“How confident do you feel in your ability to identify instigators of arguments on social media?”*.

Finally, we administered several standard demographic questions: age group, gender, education level, political ideology (1 being “very left-wing” and 7 being “very right-wing”), interest in politics (1 being “not at all interested” and 5 being “very interested”), and how often people check the news (1 being “never” and 5 being “all the time”), social media use (1 being “never” and 5 being “all the time”). Treatment group participants were also given the option to provide qualitative feedback (in a text box) about the *Cat Park* game. See Table S2 for the full sample composition.

### Supplementary Information

Supplementary Information.

## Data Availability

All data generated and analyzed in this study are included in this published article (and its Supplementary Information files). The data, supplementary information, and analysis and visualization scripts can be found on our Open Science Foundation (OSF) page: https://osf.io/ujwy3/?view_only=fe992b47176440489195b85506478d9c.
