# Supplementary material for: How to “inoculate” against multimodal misinformation: A conceptual replication of Roozenbeek and van der Linden (2020)
Source: Sci Rep. 2023 Oct 25;13:18273. doi: 10.1038/s41598-023-43885-2 (PMC10600226; doi:10.1038/s41598-023-43885-2)
Supplement: Supplementary file 1 — Supplementary Information. [file 41598_2023_43885_MOESM1_ESM.docx]

**How to “inoculate” against multimodal misinformation. A conceptual replication of Roozenbeek and van der Linden (2020)**

**Supplementary Information**

Julian Neylan,^1^, Mikey Biddlestone^2^, Jon Roozenbeek^2^, and Sander van der Linden^2^

^1^ TILT, The Hague, The Netherlands

^2^ Department of Psychology, University of Cambridge, Cambridge, United Kingdom

**Appendix A.**

**Covariate analysis**

***Exploratory linear regression model***

**Figure S1.**

*Standardised Regression Coefficients for the Mean Difference in Perceived Reliability of, Willingness to Share, and Confidence in Detecting Misinformation Between the Treatment and Control Groups, Controlling for all Covariates.*

**
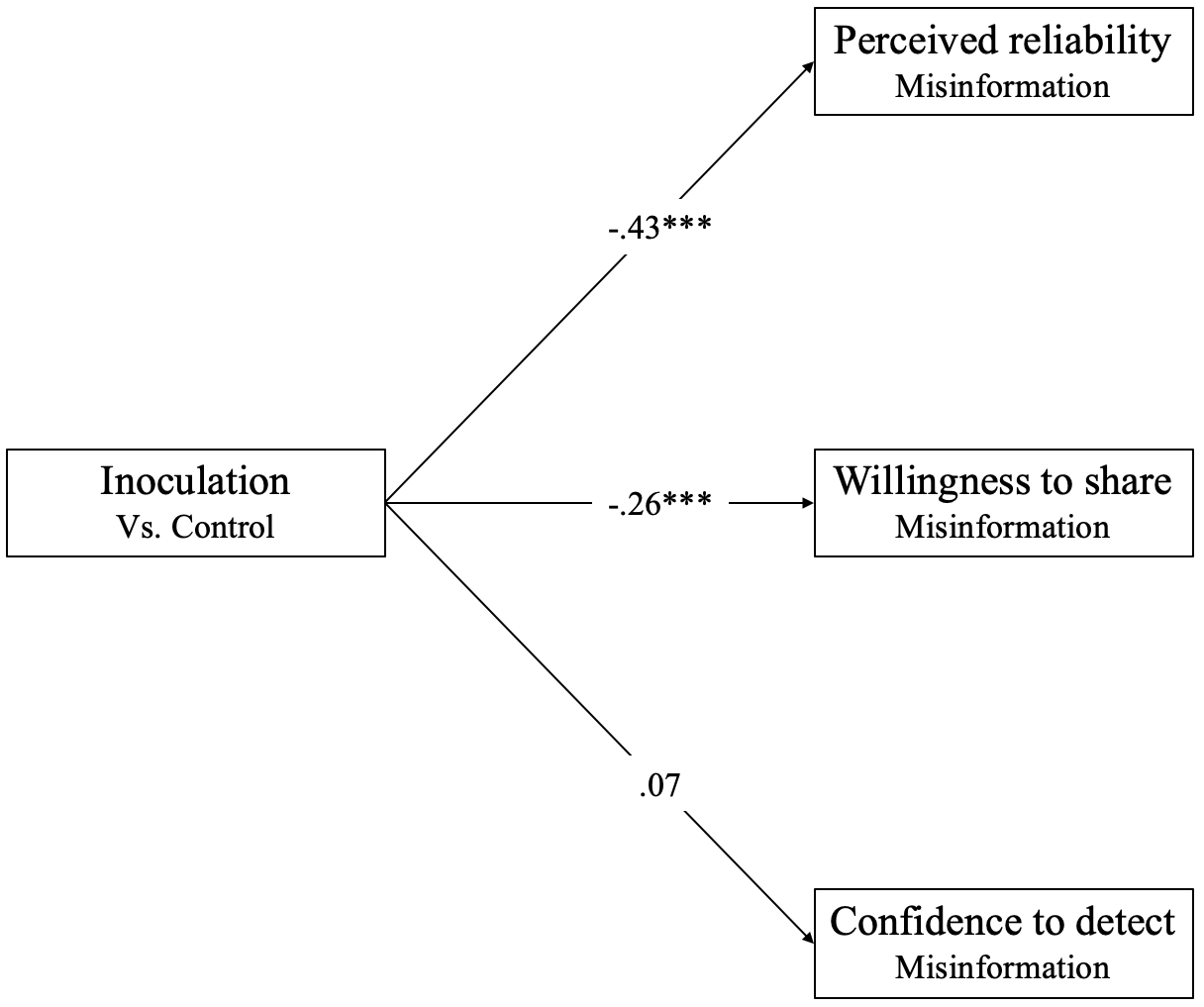
**

*Note.* *** *p* < .001.

**Table S1.**

*Standardised Regression Coefficients for the Relationships between each of the Covariates and the Outcome Variables.*

|  | Perceived reliability | Willingness to share | Confidence |
| --- | --- | --- | --- |
|  | β | β | β |
| Condition (inoculation-control) | -.43*** | -.26*** | .07 |
| Political ideology | -.02 | -.02 | -.07 |
| Political interest | -.02 | -.01 | -.01 |
| Frequency of news consumption | .05 | .03 | -.01 |
| Social media use | .01 | -.09 | .05 |
| Age | .07 | .06 | .01 |
| Gender (0 = Male, 1 = Female) | -.07 | -.11* | -.02 |
| Education | .04 | .10^☨^ | .02 |

***** = *p* < .001, ** p* = .023; ^☨^ = .052.

*Note.* regression coefficients were identical regardless of whether attention check failures were included or excluded. *N=380* regardless of whether attention check failures were included or excluded.

**Table S2.**

*Sample Composition.*

| **Variable** | *N* | % | Mean | SD |
| --- | --- | --- | --- | --- |
| **Age**       18-24       25-34       35-44       45-54       55 or older | 38  122  99  53  60 | 10.2%  32.8%  26.6%  14.2%  16.1% |  |  |
| **Gender**       Male       Female       Other | 181  194  3 | 47.6%  51.6%  0.8% |  |  |
| **Education** |  |  | 3.55 | 1.16 |
| No formal education above 16 | 31 | 8.2% |  |  |
| Prof/tech qualifications above 16 | 27 | 7.1% |  |  |
| School education up to 18 | 102 | 26.8% |  |  |
| Bachelor’s degree or equivalent | 152 | 40.0% |  |  |
| Master’s degree or equivalent | 59 | 15.5% |  |  |
| Doctorate | 9 | 2.4% |  |  |
| **Political ideology (1-7)** |  |  | 3.32 | 1.30 |
| **Political interest (1-5)** |  |  | 3.30 | 1.15 |
| **News consumption (1-5)** |  |  | 3.76 | 0.97 |
| **Social media use (1-5)** |  |  | 3.78 | 1.16 |

**Table S3.**

*Independent Samples T-Tests for all Variations of Real vs. Fake Manipulation Strategies Between Conditions*

| Independent Samples T-Test | | | | | | | | | | | | | |
| --- | --- | --- | --- | --- | --- | --- | --- | --- | --- | --- | --- | --- | --- |
|  | |  | | **Statistic** | | **df** | | **p** | |  | | **Effect Size** | |
| real.rel.diff |  | Student's t |  | -6.793 | ᵃ | 406 |  | < .001 |  | Cohen's d |  | -0.6742 |  |
| fake.rel.diff |  | Student's t |  | -7.697 | ᵃ | 378 |  | < .001 |  | Cohen's d |  | -0.7932 |  |
| real.conf.diff |  | Student's t |  | 0.243 | ᵃ | 378 |  | 0.808 |  | Cohen's d |  | 0.0250 |  |
| fake.conf.diff |  | Student's t |  | 2.136 | ᵃ | 378 |  | 0.033 |  | Cohen's d |  | 0.2202 |  |
| real.shar.diff |  | Student's t |  | -4.365 | ᵃ | 378 |  | < .001 |  | Cohen's d |  | -0.4498 |  |
| fake.shar.diff |  | Student's t |  | -4.910 | ᵃ | 378 |  | < .001 |  | Cohen's d |  | -0.5060 |  |
| real.troll.rel.diff |  | Student's t |  | -6.849 | ᵃ | 378 |  | < .001 |  | Cohen's d |  | -0.7059 |  |
| fake.troll.rel.diff |  | Student's t |  | -5.631 | ᵃ | 378 |  | < .001 |  | Cohen's d |  | -0.5803 |  |
| real.emo.rel.diff |  | Student's t |  | -4.990 | ᵃ | 378 |  | < .001 |  | Cohen's d |  | -0.5142 |  |
| fake.emo.rel.diff |  | Student's t |  | -7.401 | ᵃ | 378 |  | < .001 |  | Cohen's d |  | -0.7627 |  |
| real.conspir.rel.diff |  | Student's t |  | -6.048 |  | 378 |  | < .001 |  | Cohen's d |  | -0.6233 |  |
| fake.conspir.rel.diff |  | Student's t |  | -4.350 | ᵃ | 378 |  | < .001 |  | Cohen's d |  | -0.4483 |  |
| real.pol.rel.diff |  | Student's t |  | -4.002 | ᵃ | 378 |  | < .001 |  | Cohen's d |  | -0.4125 |  |
| fake.pol.rel.diff |  | Student's t |  | -3.320 |  | 378 |  | < .001 |  | Cohen's d |  | -0.3422 |  |
| real.troll.conf.diff |  | Student's t |  | -1.122 | ᵃ | 378 |  | 0.262 |  | Cohen's d |  | -0.1157 |  |
| fake.troll.conf.diff |  | Student's t |  | 1.096 | ᵃ | 378 |  | 0.274 |  | Cohen's d |  | 0.1129 |  |
| real.emo.conf.diff |  | Student's t |  | -0.208 | ᵃ | 378 |  | 0.836 |  | Cohen's d |  | -0.0214 |  |
| fake.emo.conf.diff |  | Student's t |  | 2.864 | ᵃ | 378 |  | 0.004 |  | Cohen's d |  | 0.2952 |  |
| real.consp.conf.diff |  | Student's t |  | 2.678 | ᵃ | 378 |  | 0.008 |  | Cohen's d |  | 0.2760 |  |
| fake.consp.conf.diff |  | Student's t |  | -0.208 |  | 378 |  | 0.836 |  | Cohen's d |  | -0.0214 |  |
| real.pol.conf.diff |  | Student's t |  | -0.541 |  | 378 |  | 0.589 |  | Cohen's d |  | -0.0557 |  |
| fake.pol.conf.diff |  | Student's t |  | 2.139 | ᵃ | 378 |  | 0.033 |  | Cohen's d |  | 0.2204 |  |
| real.troll.shar.diff |  | Student's t |  | -1.579 |  | 378 |  | 0.115 |  | Cohen's d |  | -0.1627 |  |
| fake.troll.shar.diff |  | Student's t |  | -4.514 |  | 378 |  | < .001 |  | Cohen's d |  | -0.4652 |  |
| real.emo.shar.diff |  | Student's t |  | -3.383 | ᵃ | 378 |  | < .001 |  | Cohen's d |  | -0.3487 |  |
| fake.emo.shar.diff |  | Student's t |  | -3.754 | ᵃ | 378 |  | < .001 |  | Cohen's d |  | -0.3869 |  |
| real.consp.shar.diff |  | Student's t |  | -2.942 | ᵃ | 378 |  | 0.003 |  | Cohen's d |  | -0.3032 |  |
| fake.consp.shar.diff |  | Student's t |  | -1.237 |  | 378 |  | 0.217 |  | Cohen's d |  | -0.1275 |  |
| real.pol.shar.diff |  | Student's t |  | -2.197 | ᵃ | 378 |  | 0.029 |  | Cohen's d |  | -0.2265 |  |
| fake.pol.shar.diff |  | Student's t |  | -2.705 |  | 378 |  | 0.007 |  | Cohen's d |  | -0.2788 |  |
| Note. Hₐ μ _Control_ ≠ μ _Inoculation_ | | | | | | | | | | | | | |
| ᵃ Levene's test is significant (p < .05), suggesting a violation of the assumption of equal variances | | | | | | | | | | | | | |
|  | | | | | | | | | | | | | |

**Table S4.**

*Descriptive Statistics for all Variations of Real vs. Fake Manipulation Strategies Between Conditions*

| Group Descriptives | | | | | | | | | | | | | |
| --- | --- | --- | --- | --- | --- | --- | --- | --- | --- | --- | --- | --- | --- |
|  | | **Group** | | **N** | | **Mean** | | **Median** | | **SD** | | **SE** | |
| real.rel.diff |  | Control |  | 218 |  | 0.26261 |  | 0.125 |  | 0.867 |  | 0.0587 |  |
|  | | Inoculation |  | 190 |  | 0.9178 |  | 0.750 |  | 1.079 |  | 0.0783 |  |
| fake.rel.diff |  | Control |  | 208 |  | 0.09675 |  | 0.125 |  | 0.554 |  | 0.0384 |  |
|  | | Inoculation |  | 172 |  | 0.6206 |  | 0.500 |  | 0.770 |  | 0.0587 |  |
| real.conf.diff |  | Control |  | 208 |  | -0.06791 |  | 0.000 |  | 0.504 |  | 0.0350 |  |
|  | | Inoculation |  | 172 |  | -0.0865 |  | 0.000 |  | 0.953 |  | 0.0727 |  |
| fake.conf.diff |  | Control |  | 208 |  | 0.02464 |  | 0.000 |  | 0.501 |  | 0.0347 |  |
|  | | Inoculation |  | 172 |  | -0.1265 |  | -0.125 |  | 0.859 |  | 0.0655 |  |
| real.shar.diff |  | Control |  | 208 |  | 0.05829 |  | 0.000 |  | 0.421 |  | 0.0292 |  |
|  | | Inoculation |  | 172 |  | 0.3089 |  | 0.000 |  | 0.686 |  | 0.0523 |  |
| fake.shar.diff |  | Control |  | 208 |  | 0.01262 |  | 0.000 |  | 0.374 |  | 0.0259 |  |
|  | | Inoculation |  | 172 |  | 0.2384 |  | 0.000 |  | 0.521 |  | 0.0397 |  |
| real.troll.rel.diff |  | Control |  | 208 |  | 0.09856 |  | 0.000 |  | 0.933 |  | 0.0647 |  |
|  | | Inoculation |  | 172 |  | 0.8634 |  | 0.750 |  | 1.241 |  | 0.0947 |  |
| fake.troll.rel.diff |  | Control |  | 208 |  | 0.07933 |  | 0.000 |  | 0.891 |  | 0.0618 |  |
|  | | Inoculation |  | 172 |  | 0.6715 |  | 0.500 |  | 1.158 |  | 0.0883 |  |
| real.emo.rel.diff |  | Control |  | 208 |  | 0.09856 |  | 0.000 |  | 0.840 |  | 0.0583 |  |
|  | | Inoculation |  | 172 |  | 0.5698 |  | 0.500 |  | 1.000 |  | 0.0763 |  |
| fake.emo.rel.diff |  | Control |  | 208 |  | 0.06250 |  | 0.000 |  | 0.900 |  | 0.0624 |  |
|  | | Inoculation |  | 172 |  | 0.8198 |  | 0.750 |  | 1.095 |  | 0.0835 |  |
| real.conspir.rel.diff |  | Control |  | 208 |  | -0.04567 |  | 0.000 |  | 0.817 |  | 0.0567 |  |
|  | | Inoculation |  | 172 |  | 0.4797 |  | 0.500 |  | 0.873 |  | 0.0665 |  |
| fake.conspir.rel.diff |  | Control |  | 208 |  | 0.13702 |  | 0.000 |  | 0.772 |  | 0.0535 |  |
|  | | Inoculation |  | 172 |  | 0.5320 |  | 0.500 |  | 0.997 |  | 0.0760 |  |
| real.pol.rel.diff |  | Control |  | 208 |  | 0.33173 |  | 0.000 |  | 1.026 |  | 0.0712 |  |
|  | | Inoculation |  | 172 |  | 0.8430 |  | 0.500 |  | 1.457 |  | 0.1111 |  |
| fake.pol.rel.diff |  | Control |  | 208 |  | 0.10817 |  | 0.000 |  | 0.954 |  | 0.0661 |  |
|  | | Inoculation |  | 172 |  | 0.4593 |  | 0.500 |  | 1.107 |  | 0.0844 |  |
| real.troll.conf.diff |  | Control |  | 208 |  | -0.11779 |  | 0.000 |  | 0.862 |  | 0.0598 |  |
|  | | Inoculation |  | 172 |  | 0.0145 |  | 0.000 |  | 1.412 |  | 0.1076 |  |
| fake.troll.conf.diff |  | Control |  | 208 |  | 0.03846 |  | 0.000 |  | 0.756 |  | 0.0524 |  |
|  | | Inoculation |  | 172 |  | -0.0640 |  | 0.000 |  | 1.062 |  | 0.0810 |  |
| real.emo.conf.diff |  | Control |  | 208 |  | -0.08173 |  | 0.000 |  | 0.794 |  | 0.0551 |  |
|  | | Inoculation |  | 172 |  | -0.0610 |  | 0.000 |  | 1.141 |  | 0.0870 |  |
| fake.emo.conf.diff |  | Control |  | 208 |  | 0.01683 |  | 0.000 |  | 0.912 |  | 0.0632 |  |
|  | | Inoculation |  | 172 |  | -0.2791 |  | 0.000 |  | 1.102 |  | 0.0840 |  |
| real.consp.conf.diff |  | Control |  | 208 |  | 0.00962 |  | 0.000 |  | 0.876 |  | 0.0608 |  |
|  | | Inoculation |  | 172 |  | -0.2762 |  | 0.000 |  | 1.200 |  | 0.0915 |  |
| fake.consp.conf.diff |  | Control |  | 208 |  | -0.07933 |  | 0.000 |  | 0.913 |  | 0.0633 |  |
|  | | Inoculation |  | 172 |  | -0.0581 |  | 0.000 |  | 1.077 |  | 0.0821 |  |
| real.pol.conf.diff |  | Control |  | 208 |  | -0.08173 |  | 0.000 |  | 0.898 |  | 0.0623 |  |
|  | | Inoculation |  | 172 |  | -0.0233 |  | 0.000 |  | 1.206 |  | 0.0920 |  |
| fake.pol.conf.diff |  | Control |  | 208 |  | 0.12260 |  | 0.000 |  | 0.894 |  | 0.0620 |  |
|  | | Inoculation |  | 172 |  | -0.1047 |  | 0.000 |  | 1.175 |  | 0.0896 |  |
| real.troll.shar.diff |  | Control |  | 208 |  | 0.20192 |  | 0.000 |  | 1.229 |  | 0.0852 |  |
|  | | Inoculation |  | 172 |  | 0.3895 |  | 0.000 |  | 1.054 |  | 0.0804 |  |
| fake.troll.shar.diff |  | Control |  | 208 |  | -0.08894 |  | 0.000 |  | 0.672 |  | 0.0466 |  |
|  | | Inoculation |  | 172 |  | 0.2355 |  | 0.000 |  | 0.726 |  | 0.0554 |  |
| real.emo.shar.diff |  | Control |  | 208 |  | 0.03125 |  | 0.000 |  | 0.547 |  | 0.0380 |  |
|  | | Inoculation |  | 172 |  | 0.2558 |  | 0.000 |  | 0.744 |  | 0.0568 |  |
| fake.emo.shar.diff |  | Control |  | 208 |  | -0.03125 |  | 0.000 |  | 0.706 |  | 0.0489 |  |
|  | | Inoculation |  | 172 |  | 0.2587 |  | 0.000 |  | 0.799 |  | 0.0609 |  |
| real.consp.shar.diff |  | Control |  | 208 |  | 0.08654 |  | 0.000 |  | 0.737 |  | 0.0511 |  |
|  | | Inoculation |  | 172 |  | 0.3227 |  | 0.000 |  | 0.827 |  | 0.0630 |  |
| fake.consp.shar.diff |  | Control |  | 208 |  | 0.14904 |  | 0.000 |  | 0.720 |  | 0.0499 |  |
|  | | Inoculation |  | 172 |  | 0.2471 |  | 0.000 |  | 0.825 |  | 0.0629 |  |
| real.pol.shar.diff |  | Control |  | 208 |  | 0.08654 |  | 0.000 |  | 0.666 |  | 0.0462 |  |
|  | | Inoculation |  | 172 |  | 0.2558 |  | 0.000 |  | 0.835 |  | 0.0637 |  |
| fake.pol.shar.diff |  | Control |  | 208 |  | 0.02163 |  | 0.000 |  | 0.674 |  | 0.0468 |  |
|  | | Inoculation |  | 172 |  | 0.2122 |  | 0.000 |  | 0.695 |  | 0.0530 |  |
|  | | | | | | | | | | | | | |

**Table S5.**

*Confirmatory Factor Analysis Statistics For Single-Factor Model of Perceived Reliability of Misinformation Posts*

# Confirmatory Factor Analysis

| Factor Loadings | | | | | | | | | | | |
| --- | --- | --- | --- | --- | --- | --- | --- | --- | --- | --- | --- |
| **Factor** | | **Indicator** | | **Estimate** | | **SE** | | **Z** | | **p** | |
| Factor 1 |  | real.troll.rel.diff |  | 0.572 |  | 0.0633 |  | 9.02 |  | < .001 |  |
|  |  | fake.troll.rel.diff |  | 0.622 |  | 0.0570 |  | 10.90 |  | < .001 |  |
|  |  | real.emo.rel.diff |  | 0.472 |  | 0.0521 |  | 9.05 |  | < .001 |  |
|  |  | fake.emo.rel.diff |  | 0.605 |  | 0.0574 |  | 10.53 |  | < .001 |  |
|  |  | real.conspir.rel.diff |  | 0.471 |  | 0.0484 |  | 9.74 |  | < .001 |  |
|  |  | fake.conspir.rel.diff |  | 0.476 |  | 0.0493 |  | 9.67 |  | < .001 |  |
|  |  | real.pol.rel.diff |  | 0.655 |  | 0.0692 |  | 9.47 |  | < .001 |  |
|  |  | fake.pol.rel.diff |  | 0.562 |  | 0.0566 |  | 9.93 |  | < .001 |  |
|  | | | | | | | | | | | |

## Model Fit

| Test for Exact Fit | | | | | |
| --- | --- | --- | --- | --- | --- |
| **χ²** | | **df** | | **p** | |
| 31.5 |  | 20 |  | 0.048 |  |
|  | | | | | |

| Fit Measures | | | | | | | | | | | | | | | |
| --- | --- | --- | --- | --- | --- | --- | --- | --- | --- | --- | --- | --- | --- | --- | --- |
|  | | | | | | | | **RMSEA 90% CI** | | | |  | | | |
| **CFI** | | **TLI** | | **SRMR** | | **RMSEA** | | **Lower** | | **Upper** | | **AIC** | | **BIC** | |
| 0.976 |  | 0.967 |  | 0.0313 |  | 0.0390 |  | 0.00325 |  | 0.0638 |  | 8369 |  | 8463 |  |
|  | | | | | | | | | | | | | | | |

**Table S6.**

*Confirmatory Factor Analysis Statistics For Four-Factor Model of Perceived Reliability of Misinformation Posts by Manipulation Tactic*

# Confirmatory Factor Analysis

| Factor Loadings | | | | | | | | | | | |
| --- | --- | --- | --- | --- | --- | --- | --- | --- | --- | --- | --- |
| **Factor** | | **Indicator** | | **Estimate** | | **SE** | | **Z** | | **p** | |
| Factor 1 |  | real.troll.rel.diff |  | 0.567 |  | 0.0691 |  | 8.19 |  | < .001 |  |
|  |  | fake.troll.rel.diff |  | 0.612 |  | 0.0671 |  | 9.12 |  | < .001 |  |
| Factor 2 |  | fake.emo.rel.diff |  | 0.699 |  | 0.0662 |  | 10.56 |  | < .001 |  |
|  |  | real.emo.rel.diff |  | 0.531 |  | 0.0563 |  | 9.44 |  | < .001 |  |
| Factor 3 |  | fake.conspir.rel.diff |  | 0.528 |  | 0.0548 |  | 9.63 |  | < .001 |  |
|  |  | real.conspir.rel.diff |  | 0.528 |  | 0.0540 |  | 9.78 |  | < .001 |  |
| Factor 4 |  | fake.pol.rel.diff |  | 0.533 |  | 0.0660 |  | 8.08 |  | < .001 |  |
|  |  | real.pol.rel.diff |  | 0.619 |  | 0.0790 |  | 7.83 |  | < .001 |  |
|  | | | | | | | | | | | |

## Model Fit

| Test for Exact Fit | | | | | |
| --- | --- | --- | --- | --- | --- |
| **χ²** | | **df** | | **p** | |
| 18.1 |  | 14 |  | 0.201 |  |

| Fit Measures | | | | | | | | | | | | | | | |
| --- | --- | --- | --- | --- | --- | --- | --- | --- | --- | --- | --- | --- | --- | --- | --- |
|  | | | | | | | | **RMSEA 90% CI** | | | |  | | | |
| **CFI** | | **TLI** | | **SRMR** | | **RMSEA** | | **Lower** | | **Upper** | | **AIC** | | **BIC** | |
| 0.992 |  | 0.983 |  | 0.0236 |  | 0.0278 |  | 0.00 |  | 0.0602 |  | 8367 |  | 8485 |  |
|  | | | | | | | | | | | | | | | |

**Table S7.**

*Confirmatory Factor Analysis Statistics For Two-Factor Model of Perceived Reliability of Misinformation Posts by Real vs. Fake Posts*

# Confirmatory Factor Analysis

| Factor Loadings | | | | | | | | | | | |
| --- | --- | --- | --- | --- | --- | --- | --- | --- | --- | --- | --- |
| **Factor** | | **Indicator** | | **Estimate** | | **SE** | | **Z** | | **p** | |
| Factor 1 |  | real.troll.rel.diff |  | 0.580 |  | 0.0650 |  | 8.93 |  | < .001 |  |
|  |  | real.emo.rel.diff |  | 0.474 |  | 0.0528 |  | 8.99 |  | < .001 |  |
|  |  | real.conspir.rel.diff |  | 0.479 |  | 0.0498 |  | 9.60 |  | < .001 |  |
|  |  | real.pol.rel.diff |  | 0.662 |  | 0.0704 |  | 9.40 |  | < .001 |  |
| Factor 2 |  | fake.pol.rel.diff |  | 0.567 |  | 0.0574 |  | 9.89 |  | < .001 |  |
|  |  | fake.conspir.rel.diff |  | 0.478 |  | 0.0496 |  | 9.63 |  | < .001 |  |
|  |  | fake.emo.rel.diff |  | 0.609 |  | 0.0581 |  | 10.50 |  | < .001 |  |
|  |  | fake.troll.rel.diff |  | 0.627 |  | 0.0577 |  | 10.85 |  | < .001 |  |
|  | | | | | | | | | | | |

## Model Fit

| Test for Exact Fit | | | | | |
| --- | --- | --- | --- | --- | --- |
| **χ²** | | **df** | | **p** | |
| 31.1 |  | 19 |  | 0.039 |  |
|  | | | | | |

| Fit Measures | | | | | | | | | | | | | | | |
| --- | --- | --- | --- | --- | --- | --- | --- | --- | --- | --- | --- | --- | --- | --- | --- |
|  | | | | | | | | **RMSEA 90% CI** | | | |  | | | |
| **CFI** | | **TLI** | | **SRMR** | | **RMSEA** | | **Lower** | | **Upper** | | **AIC** | | **BIC** | |
| 0.975 |  | 0.963 |  | 0.0311 |  | 0.0409 |  | 0.00915 |  | 0.0660 |  | 8370 |  | 8469 |  |
|  | | | | | | | | | | | | | | | |

**Table S8.**

*Confirmatory Factor Analysis Statistics For Single-Factor Model of Perceived Confidence of Misinformation Post Detection*

# Confirmatory Factor Analysis

| Factor Loadings | | | | | | | | | | | |
| --- | --- | --- | --- | --- | --- | --- | --- | --- | --- | --- | --- |
| **Factor** | | **Indicator** | | **Estimate** | | **SE** | | **Z** | | **p** | |
| Factor 1 |  | real.troll.conf.diff |  | 0.618 |  | 0.0612 |  | 10.10 |  | < .001 |  |
|  |  | fake.troll.conf.diff |  | 0.484 |  | 0.0488 |  | 9.92 |  | < .001 |  |
|  |  | real.emo.conf.diff |  | 0.572 |  | 0.0506 |  | 11.30 |  | < .001 |  |
|  |  | fake.emo.conf.diff |  | 0.592 |  | 0.0532 |  | 11.13 |  | < .001 |  |
|  |  | real.consp.conf.diff |  | 0.598 |  | 0.0552 |  | 10.83 |  | < .001 |  |
|  |  | fake.consp.conf.diff |  | 0.548 |  | 0.0525 |  | 10.44 |  | < .001 |  |
|  |  | real.pol.conf.diff |  | 0.604 |  | 0.0552 |  | 10.93 |  | < .001 |  |
|  |  | fake.pol.conf.diff |  | 0.579 |  | 0.0550 |  | 10.53 |  | < .001 |  |
|  | | | | | | | | | | | |

## Model Fit

| Test for Exact Fit | | | | | |
| --- | --- | --- | --- | --- | --- |
| **χ²** | | **df** | | **p** | |
| 37.1 |  | 20 |  | 0.011 |  |
|  | | | | | |

| Fit Measures | | | | | | | | | | | | | | | |
| --- | --- | --- | --- | --- | --- | --- | --- | --- | --- | --- | --- | --- | --- | --- | --- |
|  | | | | | | | | **RMSEA 90% CI** | | | |  | | | |
| **CFI** | | **TLI** | | **SRMR** | | **RMSEA** | | **Lower** | | **Upper** | | **AIC** | | **BIC** | |
| 0.971 |  | 0.959 |  | 0.0322 |  | 0.0474 |  | 0.0222 |  | 0.0709 |  | 8186 |  | 8281 |  |
|  | | | | | | | | | | | | | | | |

**Table S9.**

*Confirmatory Factor Analysis Statistics For Four-Factor Model of Perceived Confidence of Misinformation Post Detection* *by* *Manipulation Tactic*

# Confirmatory Factor Analysis

| Factor Loadings | | | | | | | | | | | |
| --- | --- | --- | --- | --- | --- | --- | --- | --- | --- | --- | --- |
| **Factor** | | **Indicator** | | **Estimate** | | **SE** | | **Z** | | **p** | |
| Factor 1 |  | real.troll.conf.diff |  | 0.516 |  | 0.0744 |  | 6.93 |  | < .001 |  |
|  |  | fake.troll.conf.diff |  | 0.416 |  | 0.0594 |  | 7.01 |  | < .001 |  |
| Factor 2 |  | fake.emo.conf.diff |  | 0.581 |  | 0.0582 |  | 9.98 |  | < .001 |  |
|  |  | real.emo.conf.diff |  | 0.562 |  | 0.0557 |  | 10.09 |  | < .001 |  |
| Factor 3 |  | fake.consp.conf.diff |  | 0.508 |  | 0.0589 |  | 8.63 |  | < .001 |  |
|  |  | real.consp.conf.diff |  | 0.567 |  | 0.0631 |  | 8.99 |  | < .001 |  |
| Factor 4 |  | fake.pol.conf.diff |  | 0.590 |  | 0.0607 |  | 9.73 |  | < .001 |  |
|  |  | real.pol.conf.diff |  | 0.607 |  | 0.0616 |  | 9.85 |  | < .001 |  |

## Model Fit

| Test for Exact Fit | | | | | |
| --- | --- | --- | --- | --- | --- |
| **χ²** | | **df** | | **p** | |
| 25.8 |  | 14 |  | 0.028 |  |
|  | | | | | |

| Fit Measures | | | | | | | | | | | | | | | |
| --- | --- | --- | --- | --- | --- | --- | --- | --- | --- | --- | --- | --- | --- | --- | --- |
|  | | | | | | | | **RMSEA 90% CI** | | | |  | | | |
| **CFI** | | **TLI** | | **SRMR** | | **RMSEA** | | **Lower** | | **Upper** | | **AIC** | | **BIC** | |
| 0.980 |  | 0.960 |  | 0.0272 |  | 0.0471 |  | 0.0155 |  | 0.0752 |  | 8187 |  | 8305 |  |
|  | | | | | | | | | | | | | | | |

**Table S10.**

*Confirmatory Factor Analysis Statistics For Two-Factor Model of Perceived Confidence of Misinformation Post Detection by Real vs. Fake Posts*

# Confirmatory Factor Analysis

| Factor Loadings | | | | | | | | | | | |
| --- | --- | --- | --- | --- | --- | --- | --- | --- | --- | --- | --- |
| **Factor** | | **Indicator** | | **Estimate** | | **SE** | | **Z** | | **p** | |
| Factor 1 |  | real.troll.conf.diff |  | 0.622 |  | 0.0618 |  | 10.07 |  | < .001 |  |
|  |  | real.emo.conf.diff |  | 0.577 |  | 0.0515 |  | 11.20 |  | < .001 |  |
|  |  | real.consp.conf.diff |  | 0.601 |  | 0.0557 |  | 10.79 |  | < .001 |  |
|  |  | real.pol.conf.diff |  | 0.607 |  | 0.0557 |  | 10.89 |  | < .001 |  |
| Factor 2 |  | fake.pol.conf.diff |  | 0.585 |  | 0.0561 |  | 10.43 |  | < .001 |  |
|  |  | fake.emo.conf.diff |  | 0.595 |  | 0.0537 |  | 11.08 |  | < .001 |  |
|  |  | fake.consp.conf.diff |  | 0.552 |  | 0.0531 |  | 10.39 |  | < .001 |  |
|  |  | fake.troll.conf.diff |  | 0.488 |  | 0.0494 |  | 9.87 |  | < .001 |  |
|  | | | | | | | | | | | |

## Model Fit

| Test for Exact Fit | | | | | |
| --- | --- | --- | --- | --- | --- |
| **χ²** | | **df** | | **p** | |
| 36.7 |  | 19 |  | 0.009 |  |

| Fit Measures | | | | | | | | | | | | | | | |
| --- | --- | --- | --- | --- | --- | --- | --- | --- | --- | --- | --- | --- | --- | --- | --- |
|  | | | | | | | | **RMSEA 90% CI** | | | |  | | | |
| **CFI** | | **TLI** | | **SRMR** | | **RMSEA** | | **Lower** | | **Upper** | | **AIC** | | **BIC** | |
| 0.970 |  | 0.955 |  | 0.0320 |  | 0.0496 |  | 0.0245 |  | 0.0734 |  | 8188 |  | 8287 |  |
|  | | | | | | | | | | | | | | | |

**Table S11.**

*Confirmatory Factor Analysis Statistics For Single-Factor Model of Sharing Intentions of Misinformation Posts*

# Confirmatory Factor Analysis

| Factor Loadings | | | | | | | | | | | |
| --- | --- | --- | --- | --- | --- | --- | --- | --- | --- | --- | --- |
| **Factor** | | **Indicator** | | **Estimate** | | **SE** | | **Z** | | **p** | |
| Factor 1 |  | real.troll.shar.diff |  | 0.345 |  | 0.0687 |  | 5.02 |  | < .001 |  |
|  |  | fake.troll.shar.diff |  | 0.282 |  | 0.0417 |  | 6.74 |  | < .001 |  |
|  |  | real.emo.shar.diff |  | 0.369 |  | 0.0369 |  | 9.98 |  | < .001 |  |
|  |  | fake.emo.shar.diff |  | 0.397 |  | 0.0438 |  | 9.08 |  | < .001 |  |
|  |  | real.consp.shar.diff |  | 0.486 |  | 0.0441 |  | 11.04 |  | < .001 |  |
|  |  | fake.consp.shar.diff |  | 0.288 |  | 0.0452 |  | 6.38 |  | < .001 |  |
|  |  | real.pol.shar.diff |  | 0.424 |  | 0.0421 |  | 10.06 |  | < .001 |  |
|  |  | fake.pol.shar.diff |  | 0.352 |  | 0.0391 |  | 8.99 |  | < .001 |  |
|  | | | | | | | | | | | |

## Model Fit

| Test for Exact Fit | | | | | |
| --- | --- | --- | --- | --- | --- |
| **χ²** | | **df** | | **p** | |
| 78.2 |  | 20 |  | < .001 |  |
|  | | | | | |

| Fit Measures | | | | | | | | | | | | | | | |
| --- | --- | --- | --- | --- | --- | --- | --- | --- | --- | --- | --- | --- | --- | --- | --- |
|  | | | | | | | | **RMSEA 90% CI** | | | |  | | | |
| **CFI** | | **TLI** | | **SRMR** | | **RMSEA** | | **Lower** | | **Upper** | | **AIC** | | **BIC** | |
| 0.861 |  | 0.806 |  | 0.0552 |  | 0.0875 |  | 0.0676 |  | 0.108 |  | 6741 |  | 6835 |  |
|  | | | | | | | | | | | | | | | |

**Table S12.**

*Confirmatory Factor Analysis Statistics For Four-Factor Model of Sharing Intentions of Misinformation Posts* *by* *Manipulation Tactic*

# Confirmatory Factor Analysis

| Factor Loadings | | | | | | | | | | | |
| --- | --- | --- | --- | --- | --- | --- | --- | --- | --- | --- | --- |
| **Factor** | | **Indicator** | | **Estimate** | | **SE** | | **Z** | | **p** | |
| Factor 1 |  | real.troll.shar.diff |  | 0.554 |  | 0.0832 |  | 6.66 |  | < .001 |  |
|  |  | fake.troll.shar.diff |  | 0.453 |  | 0.0603 |  | 7.52 |  | < .001 |  |
| Factor 2 |  | fake.emo.shar.diff |  | 0.470 |  | 0.0468 |  | 10.04 |  | < .001 |  |
|  |  | real.emo.shar.diff |  | 0.431 |  | 0.0410 |  | 10.52 |  | < .001 |  |
| Factor 3 |  | fake.consp.shar.diff |  | 0.298 |  | 0.0470 |  | 6.34 |  | < .001 |  |
|  |  | real.consp.shar.diff |  | 0.581 |  | 0.0642 |  | 9.04 |  | < .001 |  |
| Factor 4 |  | fake.pol.shar.diff |  | 0.339 |  | 0.0432 |  | 7.84 |  | < .001 |  |
|  |  | real.pol.shar.diff |  | 0.409 |  | 0.0488 |  | 8.38 |  | < .001 |  |
|  | | | | | | | | | | | |

## Model Fit

| Test for Exact Fit | | | | | |
| --- | --- | --- | --- | --- | --- |
| **χ²** | | **df** | | **p** | |
| 38.6 |  | 14 |  | < .001 |  |

| Fit Measures | | | | | | | | | | | | | | | |
| --- | --- | --- | --- | --- | --- | --- | --- | --- | --- | --- | --- | --- | --- | --- | --- |
|  | | | | | | | | **RMSEA 90% CI** | | | |  | | | |
| **CFI** | | **TLI** | | **SRMR** | | **RMSEA** | | **Lower** | | **Upper** | | **AIC** | | **BIC** | |
| 0.941 |  | 0.883 |  | 0.0390 |  | 0.0680 |  | 0.0429 |  | 0.0940 |  | 6713 |  | 6832 |  |
|  | | | | | | | | | | | | | | | |

**Table S13.**

*Confirmatory Factor Analysis Statistics For Two-Factor Model of Sharing Intentions of Misinformation Posts by Real vs. Fake Posts*

# Confirmatory Factor Analysis

| Factor Loadings | | | | | | | | | | | |
| --- | --- | --- | --- | --- | --- | --- | --- | --- | --- | --- | --- |
| **Factor** | | **Indicator** | | **Estimate** | | **SE** | | **Z** | | **p** | |
| Factor 1 |  | real.troll.shar.diff |  | 0.337 |  | 0.0686 |  | 4.92 |  | < .001 |  |
|  |  | real.emo.shar.diff |  | 0.365 |  | 0.0364 |  | 10.03 |  | < .001 |  |
|  |  | real.consp.shar.diff |  | 0.481 |  | 0.0435 |  | 11.05 |  | < .001 |  |
|  |  | real.pol.shar.diff |  | 0.416 |  | 0.0422 |  | 9.86 |  | < .001 |  |
| Factor 2 |  | fake.emo.shar.diff |  | 0.384 |  | 0.0443 |  | 8.68 |  | < .001 |  |
|  |  | fake.consp.shar.diff |  | 0.268 |  | 0.0473 |  | 5.68 |  | < .001 |  |
|  |  | fake.pol.shar.diff |  | 0.336 |  | 0.0406 |  | 8.26 |  | < .001 |  |
|  |  | fake.troll.shar.diff |  | 0.283 |  | 0.0402 |  | 7.04 |  | < .001 |  |
|  | | | | | | | | | | | |

## Model Fit

| Test for Exact Fit | | | | | |
| --- | --- | --- | --- | --- | --- |
| **χ²** | | **df** | | **p** | |
| 76.6 |  | 19 |  | < .001 |  |

| Fit Measures | | | | | | | | | | | | | | | |
| --- | --- | --- | --- | --- | --- | --- | --- | --- | --- | --- | --- | --- | --- | --- | --- |
|  | | | | | | | | **RMSEA 90% CI** | | | |  | | | |
| **CFI** | | **TLI** | | **SRMR** | | **RMSEA** | | **Lower** | | **Upper** | | **AIC** | | **BIC** | |
| 0.863 |  | 0.798 |  | 0.0549 |  | 0.0893 |  | 0.0690 |  | 0.111 |  | 6741 |  | 6840 |  |
|  | | | | | | | | | | | | | | | |

**Appendix B. Item set**

*Items (social media posts)*

| Item Name | Real-world misinformation | Fictitious misinformation |
| --- | --- | --- |
| Trolling-1 | 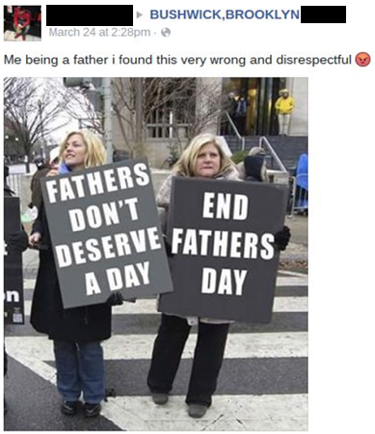 | 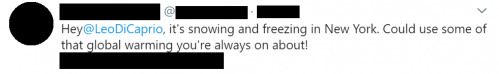 |
| Trolling-2 | 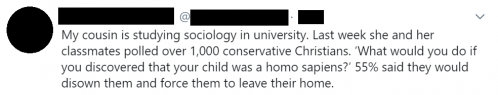 | 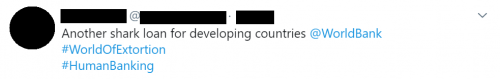 |
| Emotion-1 | 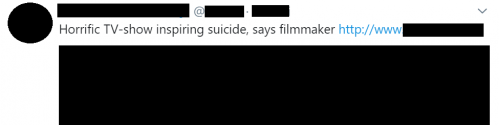 | 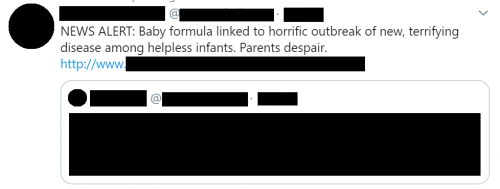 |
| Emotion-2 | 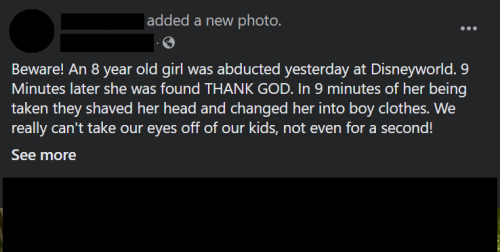 | 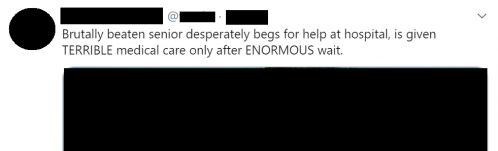 |
| Conspiracy-1 | 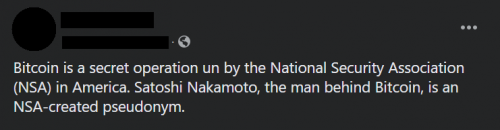 | 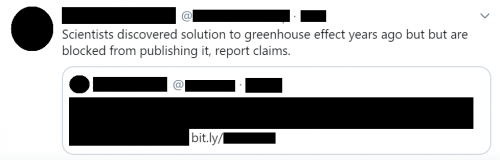 |
| Conspiracy-2 | 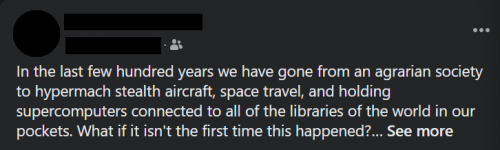 | 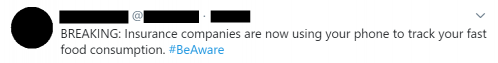 |
| Polarization-1 | 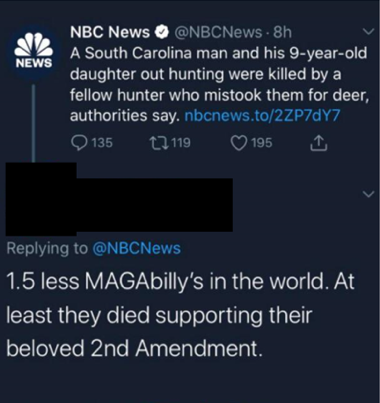 | 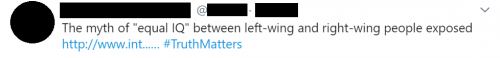 |
| Polarization-2 | 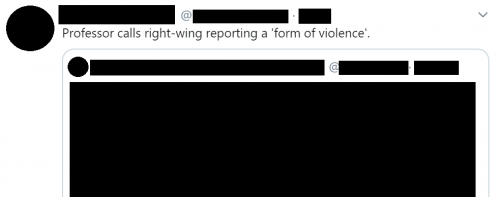 | 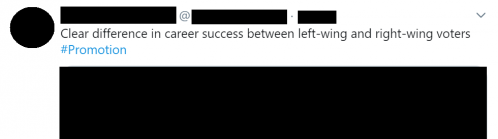 |
